# EEGgui: a program used to detect electroencephalogram anomalies after traumatic brain injury

**DOI:** 10.1186/1751-0473-8-12

**Published:** 2013-05-21

**Authors:** Justin Sick, Eric Bray, Amade Bregy, W Dalton Dietrich, Helen M Bramlett, Thomas Sick

**Affiliations:** 1 The Miami Project to Cure Paralysis, University of Miami, Miller School of Medicine, Miami, USA; 2 Department of Neurosurgery, 1095 NW 14th Terrace, Lois Pope LIFE Center, Miami, FL 33136, USA; 3 Department of Neurology, 1095 NW 14th Terrace, Lois Pope LIFE Center, Miami, FL 33136, USA; 4 Bruce W. Carter Department of Veterans Affairs, 1201 N.W. 16th Street, Miami, FL 33125, USA

## Abstract

**Background:**

Identifying and quantifying pathological changes in brain electrical activity is important for investigations of brain injury and neurological disease. An example is the development of epilepsy, a secondary consequence of traumatic brain injury. While certain epileptiform events can be identified visually from electroencephalographic (EEG) or electrocorticographic (ECoG) records, quantification of these pathological events has proved to be more difficult. In this study we developed MATLAB-based software that would assist detection of pathological brain electrical activity following traumatic brain injury (TBI) and present our MATLAB code used for the analysis of the ECoG.

**Methods:**

Software was developed using MATLAB(™) and features of the open access EEGLAB. EEGgui is a graphical user interface in the MATLAB programming platform that allows scientists who are not proficient in computer programming to perform a number of elaborate analyses on ECoG signals. The different analyses include Power Spectral Density (PSD), Short Time Fourier analysis and Spectral Entropy (SE). ECoG records used for demonstration of this software were derived from rats that had undergone traumatic brain injury one year earlier.

**Results:**

The software provided in this report provides a graphical user interface for displaying ECoG activity and calculating normalized power density using fast fourier transform of the major brain wave frequencies (Delta, Theta, Alpha, Beta1, Beta2 and Gamma). The software further detects events in which power density for these frequency bands exceeds normal ECoG by more than 4 standard deviations. We found that epileptic events could be identified and distinguished from a variety of ECoG phenomena associated with normal changes in behavior. We further found that analysis of spectral entropy was less effective in distinguishing epileptic from normal changes in ECoG activity.

**Conclusion:**

The software presented here was a successful modification of EEGLAB in the Matlab environment that allows detection of epileptiform ECoG signals in animals after TBI. The code allows import of large EEG or ECoG data records as standard text files and uses fast fourier transform as a basis for detection of abnormal events. The software can also be used to monitor injury-induced changes in spectral entropy if required. We hope that the software will be useful for other investigators in the field of traumatic brain injury and will stimulate future advances of quantitative analysis of brain electrical activity after neurological injury or disease.

## Background

The development of epilepsy is an important secondary consequence of traumatic brain injury [1-3]. Standard methods for detecting and quantifying epilepsy in an animal model include electrographic detection of epileptiform events in the brain and behavioral assessments using observer scoring systems [4]. Currently, there are a number of techniques used to quantify different phenomena associated with changes in ECoG signals. Based on a review of the current literature, the most popular methods include Power Spectral Density (PSD), Short Time Fourier analysis and Spectral Entropy (SE). For our study, we built a graphical user interface (GUI) in the MATLAB programming platform that allows the user to analyze ECoG signals using the three methods mentioned above. In this paper we present our methods and MATLAB code used for the analysis of ECoG signals.

The basic PSD of a signal x(t_i_) describes how the power is distributed with respect to frequency. It is calculated by multiplying the Fast Fourier Transform (FFT) by the complex conjugate of the Fourier component.

$$P\left(f_{i}\right)=X\left(f_{i}\right)\times X^{*}\left(f_{i}\right)$$

Where

$$X\left(f_{i}\right)=\sum_{t_{i}}x\left(t_{i}\right)e^{-2\mathrm{\pi i}f_{i}t_{i}}$$

and X^*^ (*f*_*i*_) is the complex conjugate of the Fourier component *X* (*f*_*i*_) [5].

The PSD is very effective for describing which frequency components of a signal are most prominent. Unfortunately, there is no time information when signals are processed this way. For a signal that is changing over time, this method lacks the ability to identify when specific frequency components are present in the signal.

The Short Time Fourier Transform (STFT) advances through the signal in epochs. Each epoch is transformed individually which allows for frequency analysis in time domain. This is essential for linking specific events seen in the ECoG signal to changes in specific frequency components.

Different frequency components are associated with unique brain functions or pathologies. Delta rhythms are normally defined to be between frequencies 0.5-3.5Hz and are characteristic of deep sleep stages. Frequencies between 3.5-7.5Hz are called theta rhythms and are enhanced by the limbic system. Alpha rhythms (7.5-12.5Hz) are most prominent during drowsiness or coma, beta rhythms (12.5-30Hz) are enhanced with alertness and gamma rhythms (30-60Hz) are linked to common perceptual information [6]. We refer to neurophysiology literature for more information on the different frequency bands since this is not the focus of this manuscript.

Spectral Entropy describes the order of a signal in terms of power distributed across a range of frequencies. In this context, entropy describes the irregularity, complexity, or unpredictability characteristics of a signal [5]. The process for calculating SE from a signal x(t_i_) is given by [5] and is as follows.

First, the power spectrum is normalized so that the sum of the normalized power spectrum over the selected frequency range is equal to one.

$$\sum_{f_{i}=f_{2}}^{f_{2}}\mathrm{Pn}\left(f_{i}\right)=C_{n}\sum_{f_{i}f_{1}}^{f_{2}}P\left(f_{i}\right)=1$$

SE is then calculated as the sum,

$$S\left[f_{1}f_{2}\right]=\sum_{f_{i}=f_{1}}^{f_{2}}〚P_{n}\left(f_{i}\right)\log\left(\frac{1}{P_{n}\left(f_{i}\right)}〛\right)$$

The SE is then normalized to a range between 1 (maximum irregularity) and 0 (complete regularity). This is done by dividing by the factor log of the total number of frequency components in the selected frequency range, N[f_1_,f_2_].

$$S_{N}=\frac{S\left[f_{1},f_{2}\right]}{\log\left(N\left[f_{1},f_{2}\right]\right)}$$

## Methods

### Animals and ECoG recordings

All procedures involving the use of live animals for this study were reviewed and approved by the Institutional Animals Care and Use Committee at the University of Miami School of Medicine. In this study we monitored ECoG activity and behavior (by video recording) simultaneously in awake freely moving rats 1 year after moderate (2.0 ATM) parasagittal fluid percussion brain injury (mTBI) or sham injury [2]. Twenty four hours prior to recording, animals were anesthetized with isofluorane (1.5%) and silver-silver choride electrodes were implanted over the dura mater through 1 mm burrholes in the skull. The electrodes were fixed in place with dental acrylic. Twenty four hours after recovery from surgery the electrodes were attached to a custom designed first stage amplifier (gain X 100, 0.1 Hz- 1 KHz bandpass filtered) and the headstage amplifier was tethered to a second stage amplifier by means of a flexible cable. Signals were amplified again and further filtered (final gain X 500, bandpass 0.1Hz – 500 Hz). Analog signals were digitized and stored using AD Instruments Powerlab™ and LabChart software.

### EEG seizure analysis

**Figure 1:**
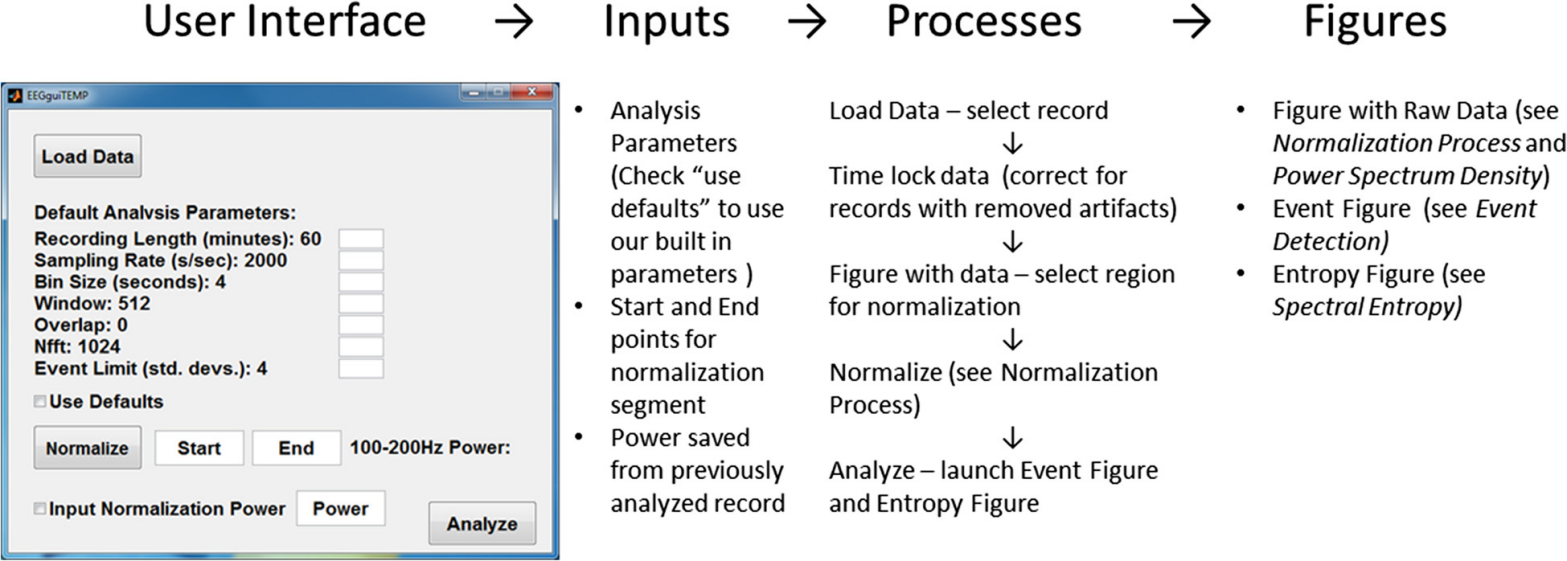
Visual outline of the steps taken by EEGgui to analyze an ECoG record.

The overall goal of this analysis is to detect abnormal events in the ECoG recordings that are periodic and not seen or seen less frequently in recordings from naïve animals. This was accomplished by: 1) normalizing power density to reduce variability that might occur between recordings due to differences in signal quality (ECoG amplitude) and 2) by detecting events over time defined as power density values that exceeded those found in a predefined segment of “normal” ECoG activity. Our EEG analysis software (EEGgui) was developed by modifying EEGLAB open access software running on the MATLAB platform. EEGgui offers researchers who are not proficient in computer programming to run analysis on ECoG signals using the MATLAB programming platform. The flow of data import, processing and figure production is shown in Figure 1.

### Import data

All files are imported as text files which allows for signals to be analyzed independent of the recording software. Records should have two dimensions, the voltage data and a time component. Our import function advances through the record looking for discontinuities in the time array based on the sampling frequency (Fs), then fills in missing data with NaN (not a number) values to keep the time component synched with the voltage data in signals that have segments removed due to artifacts. This is especially important for studies tracking changes in ECoG over time after a stimulus. The function (timelock.m) is provided in Additional file 1 along with detailed comments.

**Figure 2:**
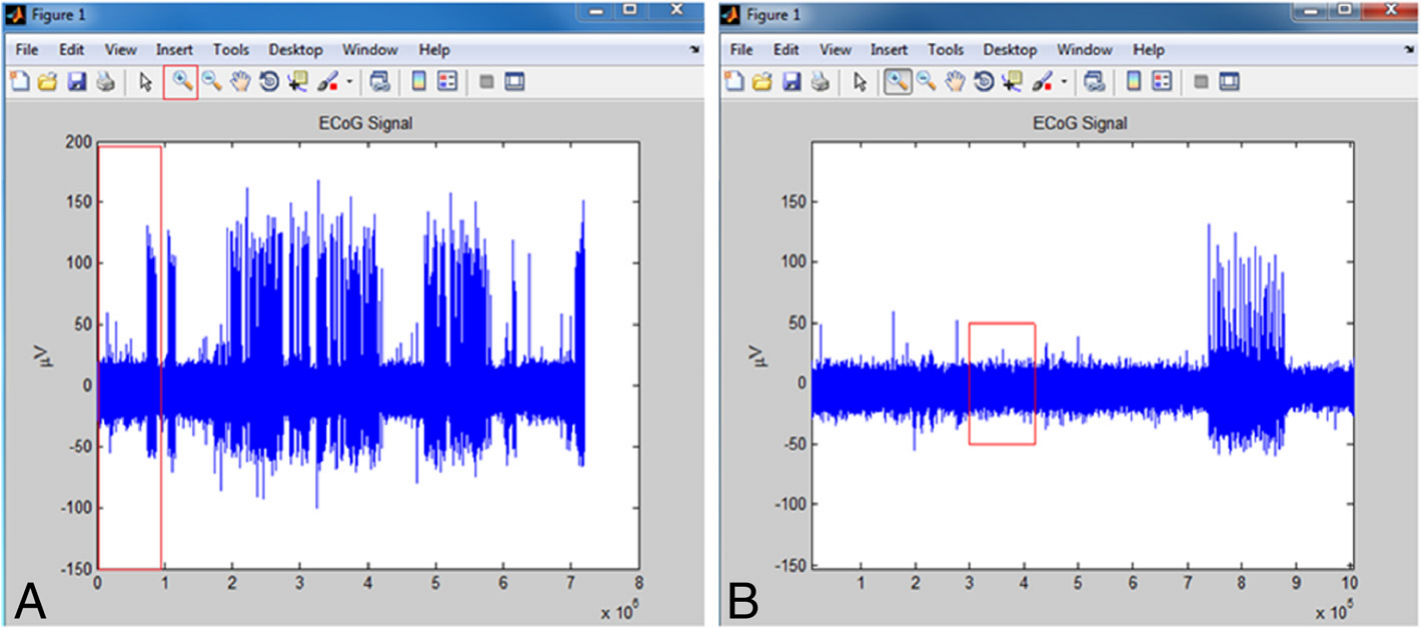
**Screen shot of the raw data figure returned from EEgui. A**. Initial Figure launched with raw data. The user can use the built in zoom function to find a region of awake ECoG. **B**. Zoomed in to region of interest (red area from **A**). The x-axis is in total data points, not time, for this figure. We suggest selecting 1 minute of ECoG. From our notes used during observation, we know the animal was awake and freely moving until the 5 minute mark which is equivalent to the 600,000^th^ point, recording at 2000 samples per second. Therefore, for 1 minute of behavior, we select a start point of 300,000 and endpoint of 420,000 (red area in **B**).

One constraint is that the record must be continuous. If the time component of the imported file does not advance in one direction throughout the record, an error will occur. The most common cause of this error when recording with LabChart is if the record is stopped and restarted, resulting in the time returning to 0 at some point during the record. Exampales of imported ECoG data are shown in Figure 2.

### Power normalization

One issue to overcome when analyzing ECoG in frequency domain is the dependence of power on signal strength. It is known that signal strength can vary depending on the distance of the electrodes from the source potential (brain tissue) and the conductivity of the tissue. While the amplitude of the recorded signal might not appear to vary significantly among animals, small changes in amplitude result in larger changes in power as power is a function of the square of the signal amplitude. Therefore, to minimize this potential problem we first select a region of the signal that is associated with awake, exploratory behavior of rats. We then average the power for a frequency range outside the range of interest (e.g. 100Hz-200Hz) and divide all powers by this value. For users with recording systems having more limited bandpass, the software must be modified to normalize power using a frequency band within recording limits (a description of this modification is provided with the source code.

The normalization procedure operates under two debatable assumptions. The first is that all differences in signal amplitude at the normalization frequency will be reflected by proportional changes in amplitude at the frequencies of interest. For example, in the case where signal amplitude (in microvolts) depends on the distance from the recording electrodes to the signal source (brain) we assume that the difference in amplitude of the signal at the normalization frequency will be proportional to the difference in amplitude at the frequencies of interest. In other words, assume a signal contains two frequencies *f*_*1*_ and *f*_*2*_. The measured amplitudes will be, *f*_*1*_*(x)* and *f*_*2*_*(x)* for a distance x from the signal source. If the signal source is then moved to a distance *x +* Δ*x,* the frequencies will have amplitudes of *f*_*1*_*(x +* Δ*x)* and *f*_*2*_*(x +* Δ*x)*. Under our assumption,

$$\frac{f_{1}\left(x\right)}{f_{2}\left(x\right)}=\frac{f_{1}\left(x+\mathrm{\Delta x}\right)}{f_{2}\left(x+\mathrm{\Delta x}\right)}$$

Our second assumption is that ECoG at 100-200Hz measured during periods of awake, exploratory behavior is minimally affected by TBI. This will be discussed further below.

### Power spectrum density

Our PSDs are calculated using Welch’s method. A detailed explanation of Welch’s method can be found in the MATLAB help. The *pwelch* function is available in the MATLAB signal processing toolbox, if the signal processing toolbox is not available, Lanspeary © 2006 developed a function that is available via the Free Software Foundation and is provided in our code.

**Figure 3:**
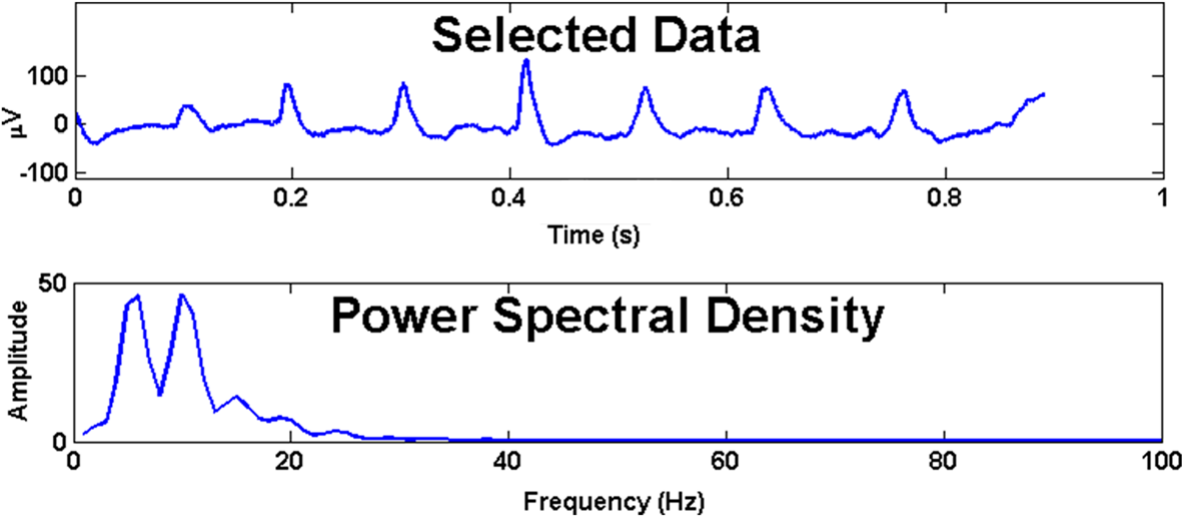
**Selected region for PSD analysis from Figure **2**a.** User can use the built in zoom function in MATLAB to narrow in on a region of interest in the raw signal record. Click “PSD,” then select region and this figure will appear which includes the raw data record (top trace) and the power density versus frequency graph (bottom trace).

We allow the user to select a region of interest on a plot of the signal to evaluate. This is a simple and efficient way to deduce the frequency components of particular segments of a signal corresponding to different behavior. An example of power spectral analysis from a data sample is shown in Figure 3.

### Event detection

The STFT is used in part as our “event detection.” As mentioned earlier, the STFT is a Fourier transform performed on short time epochs of a signal. Editable variables in this part of the analysis are the length of each epoch and standard deviations. We define the frequency bands delta, theta, alpha, beta I, beta II and gamma and identify events where the experimental power deviates from control power within a frequency band.

Before events can be detected, the average power and standard deviations for each frequency band must be calculated from the control group to be compared against. Similar to our normalization, we use a 1 minute segment of awake, exploratory behavior. In the code we provide our calculated values. Included in the Additional file 1 is a detailed explanation on how to store new values if the user wishes to include their own recordings.

**Figure 4:**
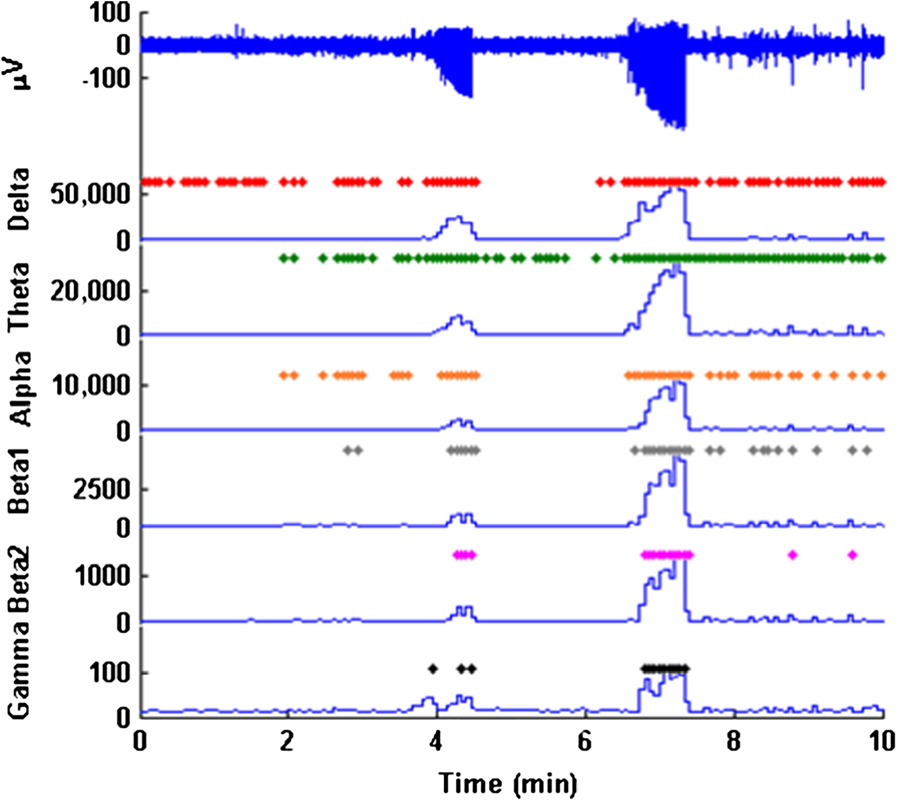
**First 10 minutes of recording after injection of pentylene tetrazol (PTZ).** The two high amplitude bursts near 4 and 7 min are seizures. These are detected as events with power increases in all frequency band. In contrast, following the second seizure ECoG events associated with sleep are detected in lower frequency bands.

The user then inputs the number of standard deviations the experimental group power must deviate from the control power necessary to trigger an event. The program uses this information to proceed through the signal in steps, calculating the mean power for each frequency band and identifying events with dots. A figure will appear with the raw signal and power plots with events marked by dots. An example is shown in Figure 4.

After each signal is analyzed, a variable is saved to the MATLAB workspace that holds the event data for each frequency band. The number of events for each recording can then be compared between all groups. For statistical analysis outside of MATLAB, event data can be copied to Microsoft EXCEL or a familiar software package.

### Spectral entropy

**Figure 5:**
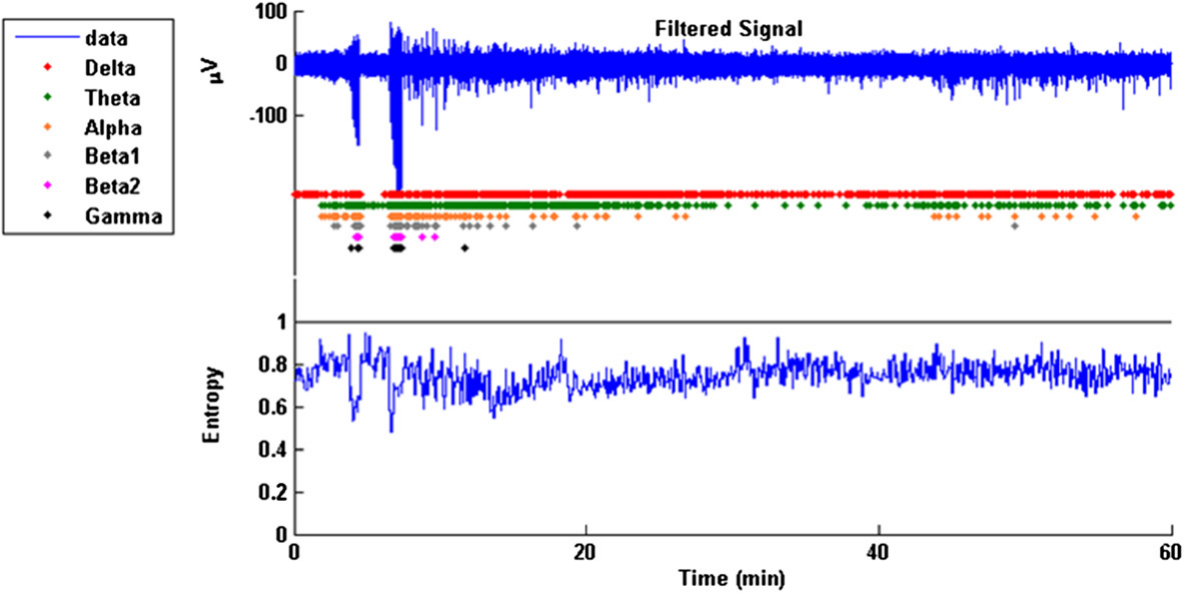
**Spectral Entropy (lower panel) plotted along with the filtered ECoG signal (top trace) and event detection as described above.** The trace shows two seizure events followed by sleeping. The animal’s behavior was confirmed by visual observation and video analysis.

During periods of awake, exploratory behavior, the entropy will be greatest because of the activity which is present across all frequency bands. During synchronous epileptic discharges, the majority of power is expressed at fewer frequencies, therefore the entropy will decrease. The spectral entropy is normalized to be independent of power, therefore we do not have to normalize each individual animal, instead we ratio it to our segment of normal behavior from naïve animals. No values much greater than one should be detected because of the assumption that normal behavior has the greatest entropy. Values of less than one represent segments of the signal where the SE has decreased. An example is shown in Figure 5 using a longer segment of the same data from which Figure 4 was derived.

Before SE is calculated, the signal is low pass filtered at 60Hz. The filter can be found in the Additional file 1. This allows removal of electromyographic (EMG) contamination of the SE calculation during seizures. Powers in EMG frequencies diminish the changes in SE by spreading power across a wide range of frequencies that we are not studying. The NaN values used in the loading process are replaced with zeros for the filtering calculation then converted back to NaN before calculating SE.

## Results

**Figure 6:**
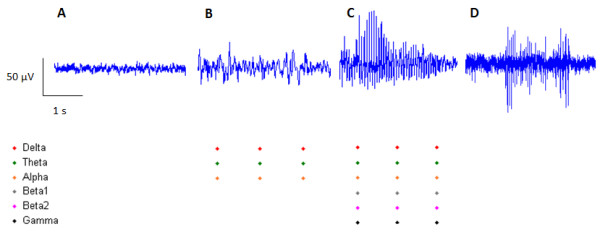
**Examples of ECoG recordings from an animal during awake exploratory behavior, sleeping, seizure activity, and chewing (electromyographic (EMG) artifact).** Significant changes in spectral power for 2sec intervals are indicated by dots for the respective frequencies. Note that no significant events are detected for ECoG collected during awake behavior (as expected since this is the comparator signal) or during chewing artifact since EMG frequencies are outside of our selected power bands. Significant events are detected at slow frequencies during sleep and across all frequencies during seizures.

We were able to distinguish between a variety of behaviors represented in the ECoG signal such as awake and exploratory behavior, muscle artifact, sleep and different seizure categories. Examples of ECoG traces and detected events in the different power bands are shown in Figure 6.

The ECoG during awake periods is of small amplitude. No peaks in the PSD are seen, no events in any power band are recorded and the SE is at its greatest during awake, exploratory behavior.

Muscle artifact had no affect on our analysis. Large bursts in the signal could be seen when the animal was chewing and grinding teeth. The power in EMG is expressed at frequencies higher than the power in ECoG and therefore not seen on PSD comparisons. This was confirmed using our event detection where no events were recorded using any of the frequency bands analyzed. Changes in SE are also not seen because the signal is filtered above 60Hz.

Sleeping was identified as a single peak in the PSD below 10Hz. The delta, theta, and alpha bands most often triggered events with the beta I and beta II bands occasionally triggering events during periods of sleep. The gamma band power does not trigger an event during sleep. SE is decreased during periods of sleep.

Seizures showed a massive increase in power across the frequency spectrum. During periods of epileptic activity, events were recorded in each frequency band. Spectral Entropy decreased dramatically.

## Discussion

In this paper, we have described a program called EEGgui that was developed in the MATLAB programming platform. The purpose of the program is to allow users to quantify changes in brain activity compared to a pre-defined behavioral and electrographic state (in our case the awake state). ECoG records were used to investigate the onset of epilepsy 1 year after TBI. We put together an interface that includes the most common calculations currently used and introduced new concepts such as our event detection and the normalization process.

Among the many conditions associated with TBI, disturbances in sleeping patterns and frequent periods of fatigue are extremely common. A recent review of TBI and sleep disturbance suggests the need for objective, measurable deficits in sleep [7,8]. To study sleeping patterns, long term observation under standard living conditions is necessary. Subjects would not need to be kept under strict observation for the entire recording period, a few lines of code could total the number of delta events occurring by themselves and compare these findings to control groups. Fatigue could be studied in a similar manner by examining the number of events in the alpha band.

The use of power in the 100–200 Hz band for the normalization process has yet to be proven. There are at least two known events that can occur in this frequency range in awake, freely moving animals. The first is muscle contraction artifact and the second is high frequency oscillations known as “ripples” [9]. There is literature that suggests the development of ripples in the 100–200 Hz range are associated with epilepsy and thus might occur after TBI [10-12]. Both are periodic and recognizable through spectra analysis. As a result, they can be easily avoided by selecting short records of ECoG devoid of these components for normalization. Normalization might not be necessary if the raw ECoG signals among subjects are identical. However, power is a function of the square of ECoG amplitude and thus small differences in raw signal amplitude among subjects will result in large differences in power.

As mentioned above, the FFT provides no time information from the original signal. This is improved by advancing through the signal in short epochs, taking the FFT of each individual time segment. This method is still not perfect as it is limited by the uncertainty principle. If the epochs are too short, slow frequencies cannot be resolved. If epochs are too long, the specific time in which frequencies are present is lost.

Another transform to be considered which may be an improvement to the STFT is the Wavelet Transform. The Wavelet Transform has a varying epoch, a long epoch to accommodate slow frequencies and a short epoch for fast frequencies. Because the wavelet transform does not require a fixed epoch, it has advantages over STFT for signals with features that vary significantly in duration and frequency, such as ECoG [13]. This transform could prove to be a substantial improvement for our event detection and entropy analysis. MATLAB also provides a Wavelet toolbox that could be implemented into ECoG analysis. We are currently working on introducing a wavelet analysis package into our GUI.

As our study progresses, we expect to use these calculations to automate behavior recognition through ECoG analysis. An automated process will allow us to efficiently quantify changes in sleep patterns and epileptic activity, as well as identify other changes which might not be currently known to be associated with TBI.

## Abbreviations

EEG: Electroencephalography; ECoG: Electrocorticographic; TBI: Traumatic brain injury; PSD: Power Spectral Density; SE: Spectral Entropy; GUI: Graphical user interface; FFT: Fast Fourier Transform; STFT: Short Time Fourier Transform; 2.0 ATM: Moderate; mTBI: Parasagittal fluid percussion brain injury; Fs: Sampling frequency; NaN: Not a number; PTZ: Pentylene tetrazol; EMG: Electromyographic.

## Competing interests

The authors declare that they have no competing interests.

## Authors’ contributions

JS and EB contributed equally to the design of the EEGgui software, including code for entering data from the user interface, code for normalization, code for event detection, and code for data displays. AB performed the animal injuries, implantation of recording devices, PTZ injections, contributed to the development of recording hardware and video analysis and participated in the design of the study. DD and HB provided the original experimental design for production of TBI in animals used in this study and they also provided assistance with interpretation of injury severity. TS was responsible for design of ECoG procedures, assurance of the quality of ECoG recordings, interpretation of ECoG signals, and interpretation of the validity of measures returned by EEGgui software. All authors read and approved the final manuscript.

## Supplementary Material

Additional file 1 Timelock.m: a function that places Not A Number (NaN) in place of missing data. Click here for file
