# Supplementary material for: EEGgui: a program used to detect electroencephalogram anomalies after traumatic brain injury
Source: Source Code Biol Med. 2013 May 21;8:12. doi: 10.1186/1751-0473-8-12 (PMC3673894; doi:10.1186/1751-0473-8-12)
Supplement: Additional file 1 — Timelock.m: a function that places Not A Number (NaN) in place of missing data. [file 1751-0473-8-12-S1.zip › 2002468826834285_add26.pptx]

## Slide 1
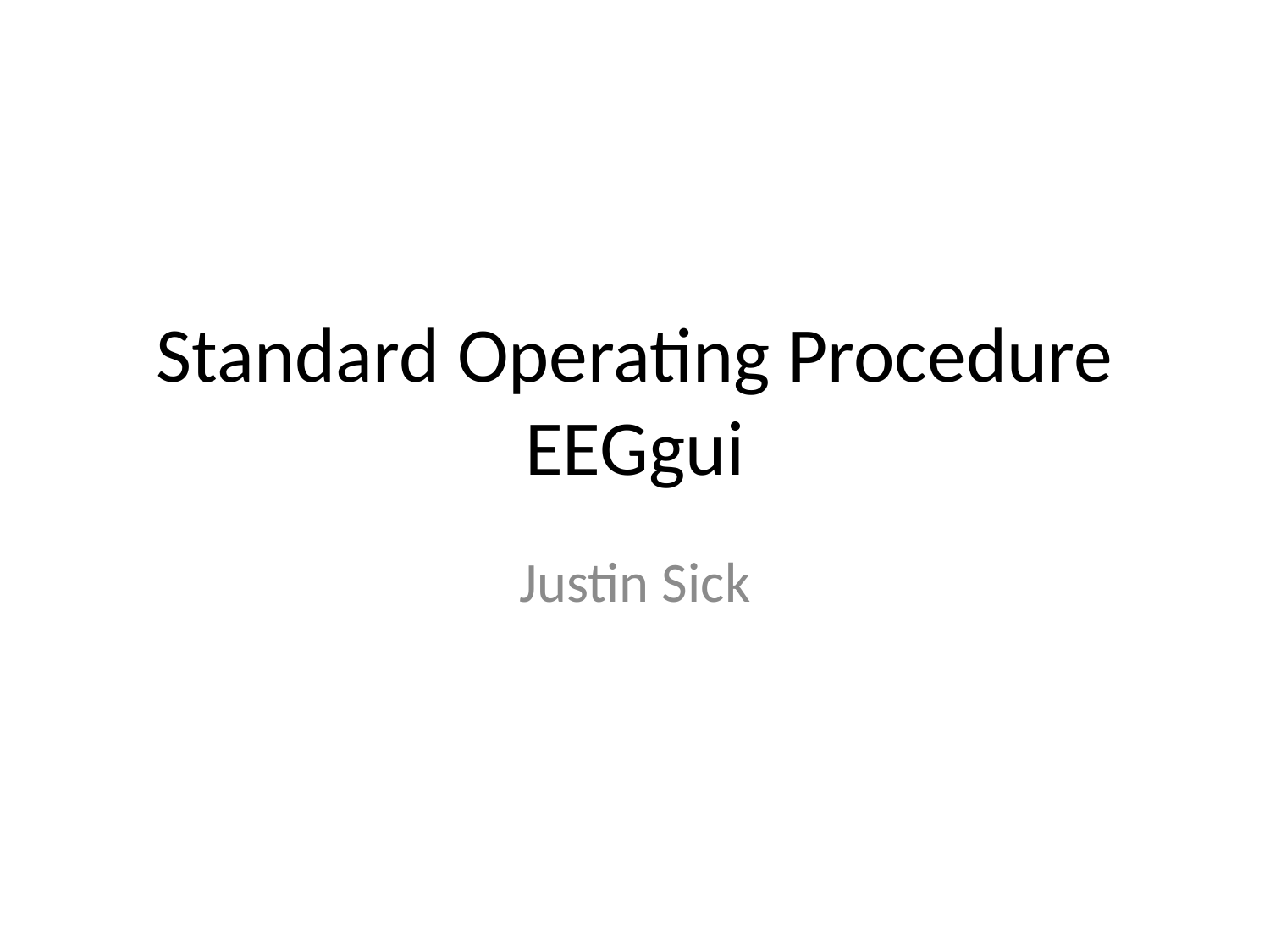

# Standard Operating Procedure EEGgui
Justin Sick

## Slide 2
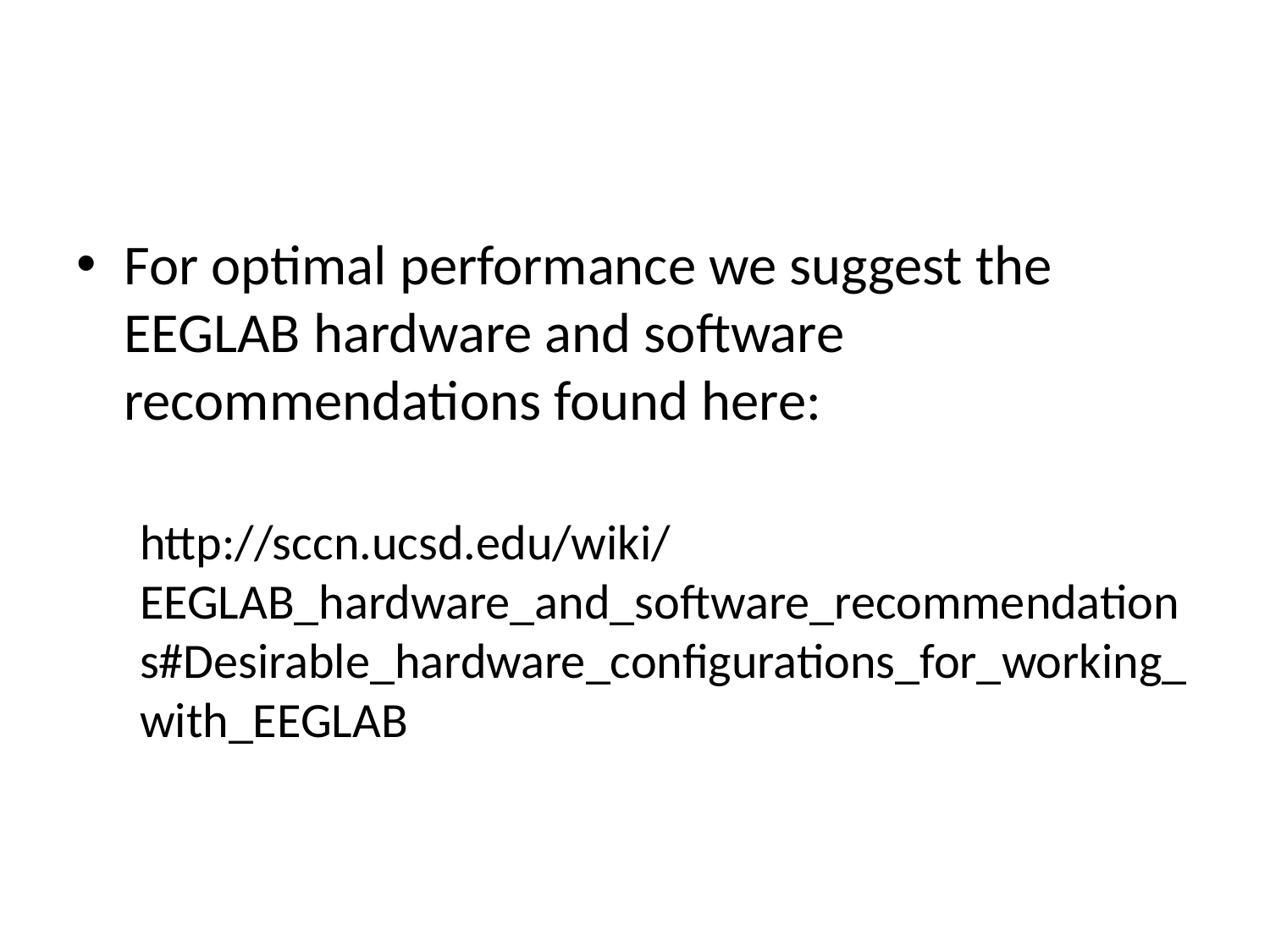

For optimal performance we suggest the EEGLAB hardware and software recommendations found here:
http://sccn.ucsd.edu/wiki/EEGLAB_hardware_and_software_recommendations#Desirable_hardware_configurations_for_working_with_EEGLAB

## Slide 3
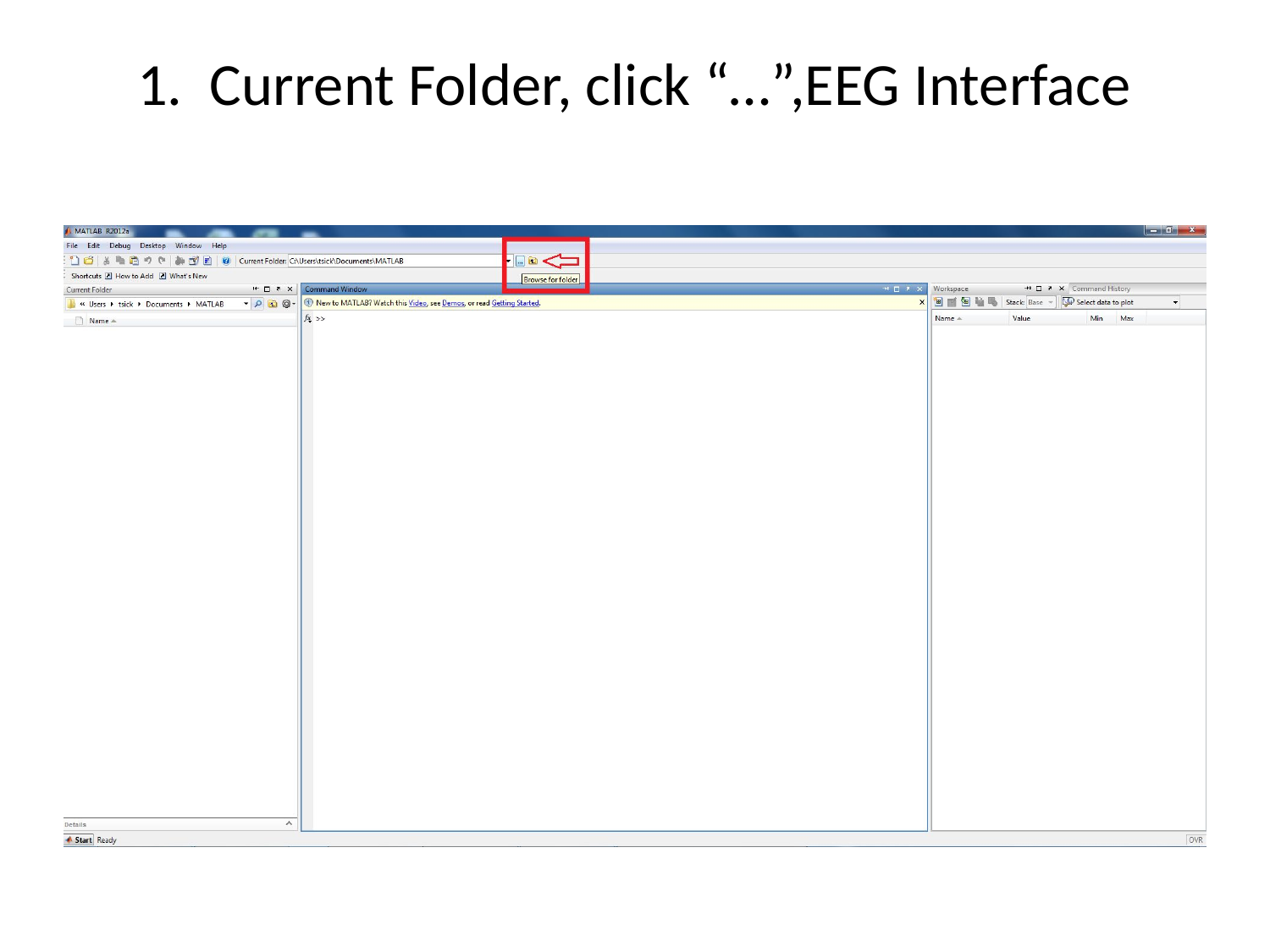

# 1. Current Folder, click “…”,EEG Interface

## Slide 4
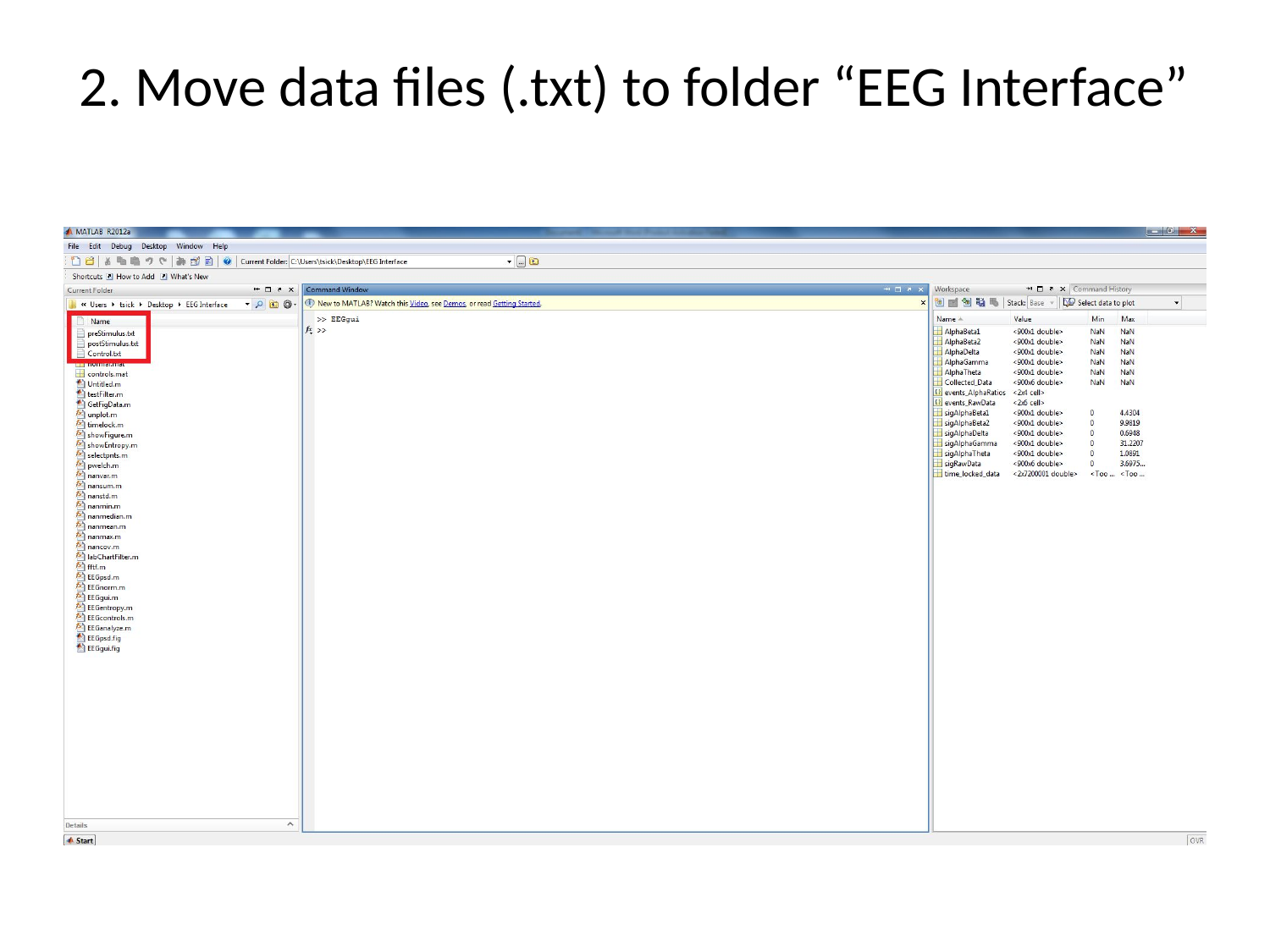

# 2. Move data files (.txt) to folder “EEG Interface”

## Slide 5
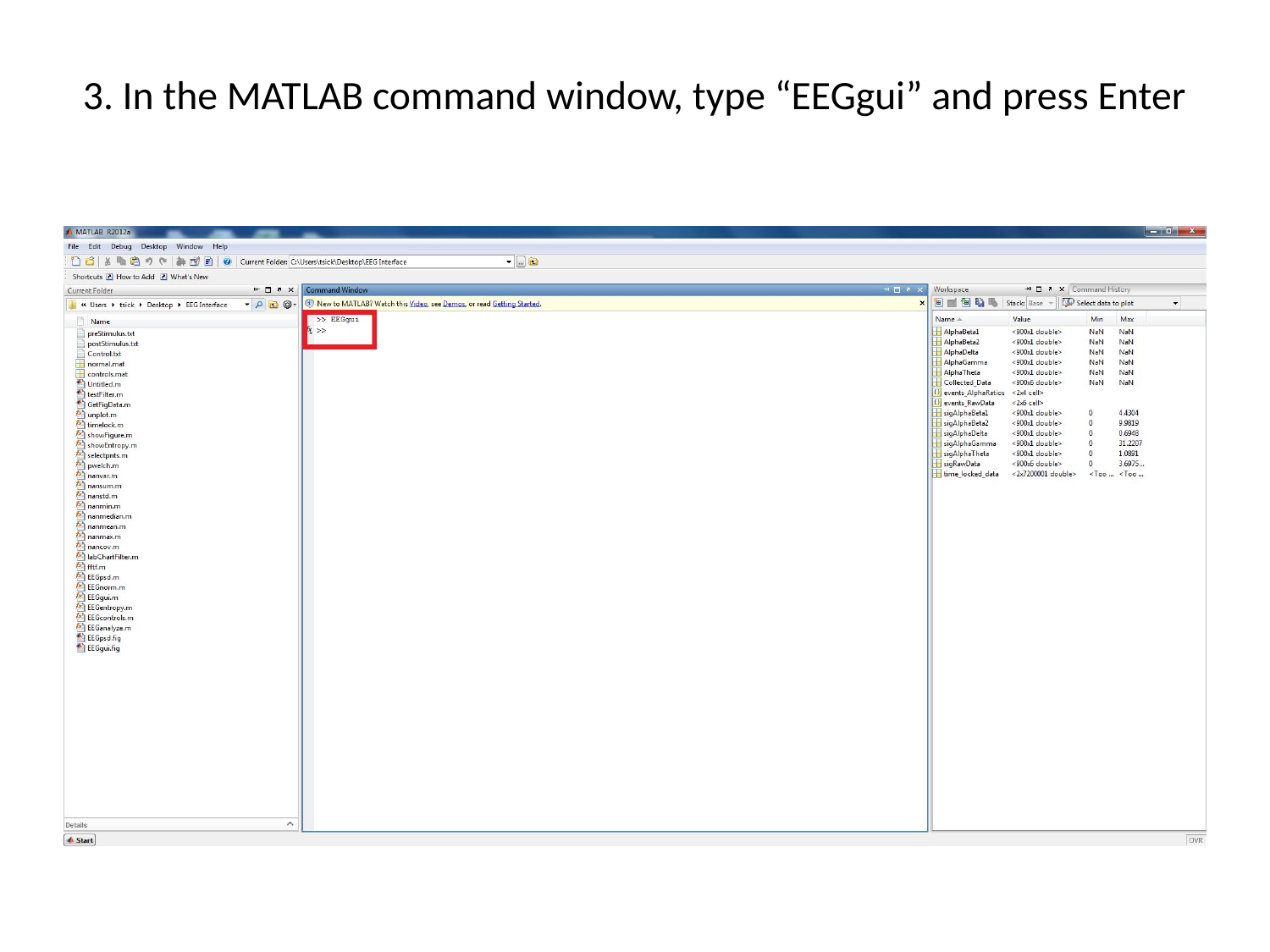

# 3. In the MATLAB command window, type “EEGgui” and press Enter

## Slide 6
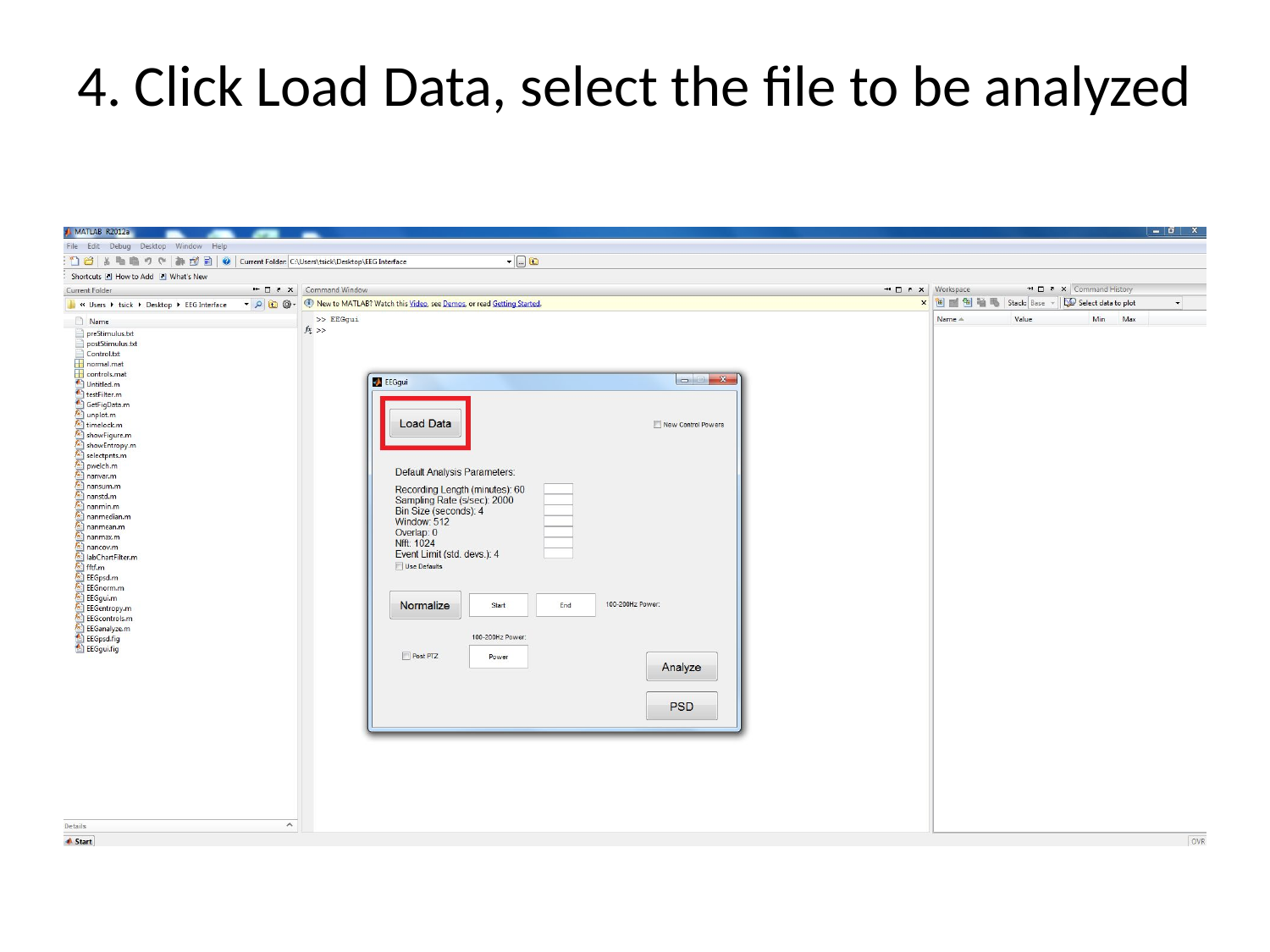

# 4. Click Load Data, select the file to be analyzed

## Slide 7
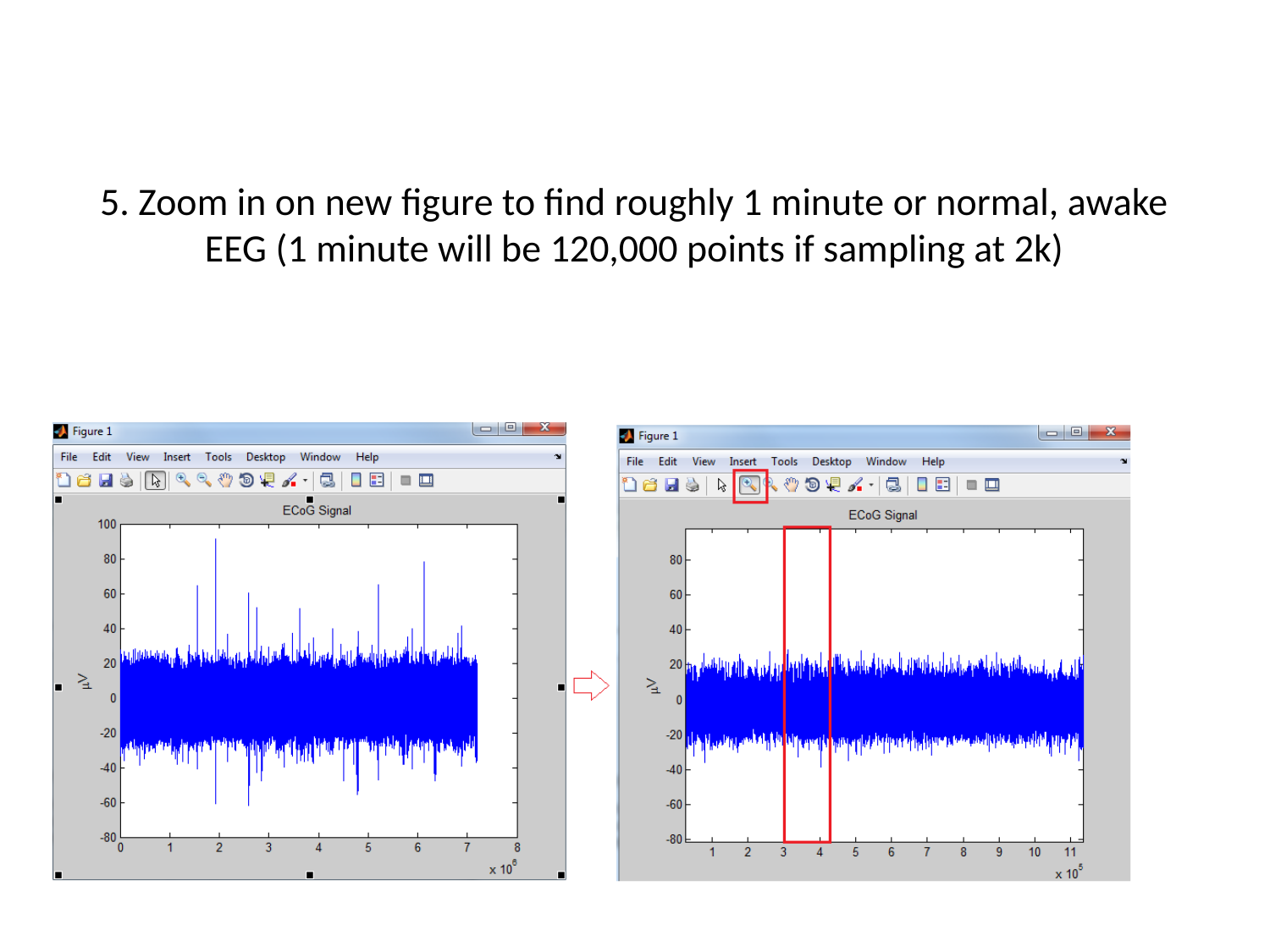

# 5. Zoom in on new figure to find roughly 1 minute or normal, awake EEG (1 minute will be 120,000 points if sampling at 2k)

## Slide 8
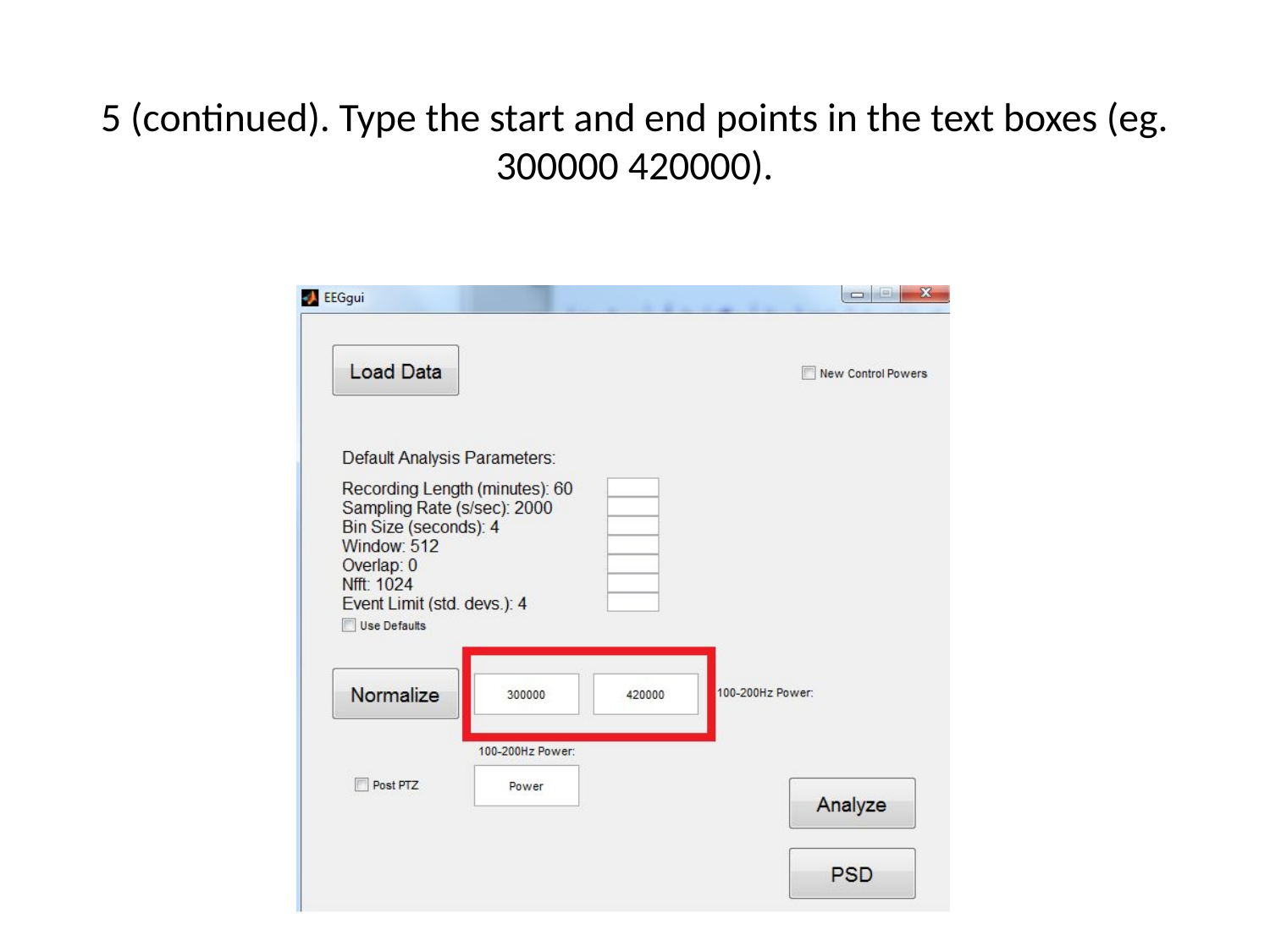

# 5 (continued). Type the start and end points in the text boxes (eg. 300000 420000).

## Slide 9
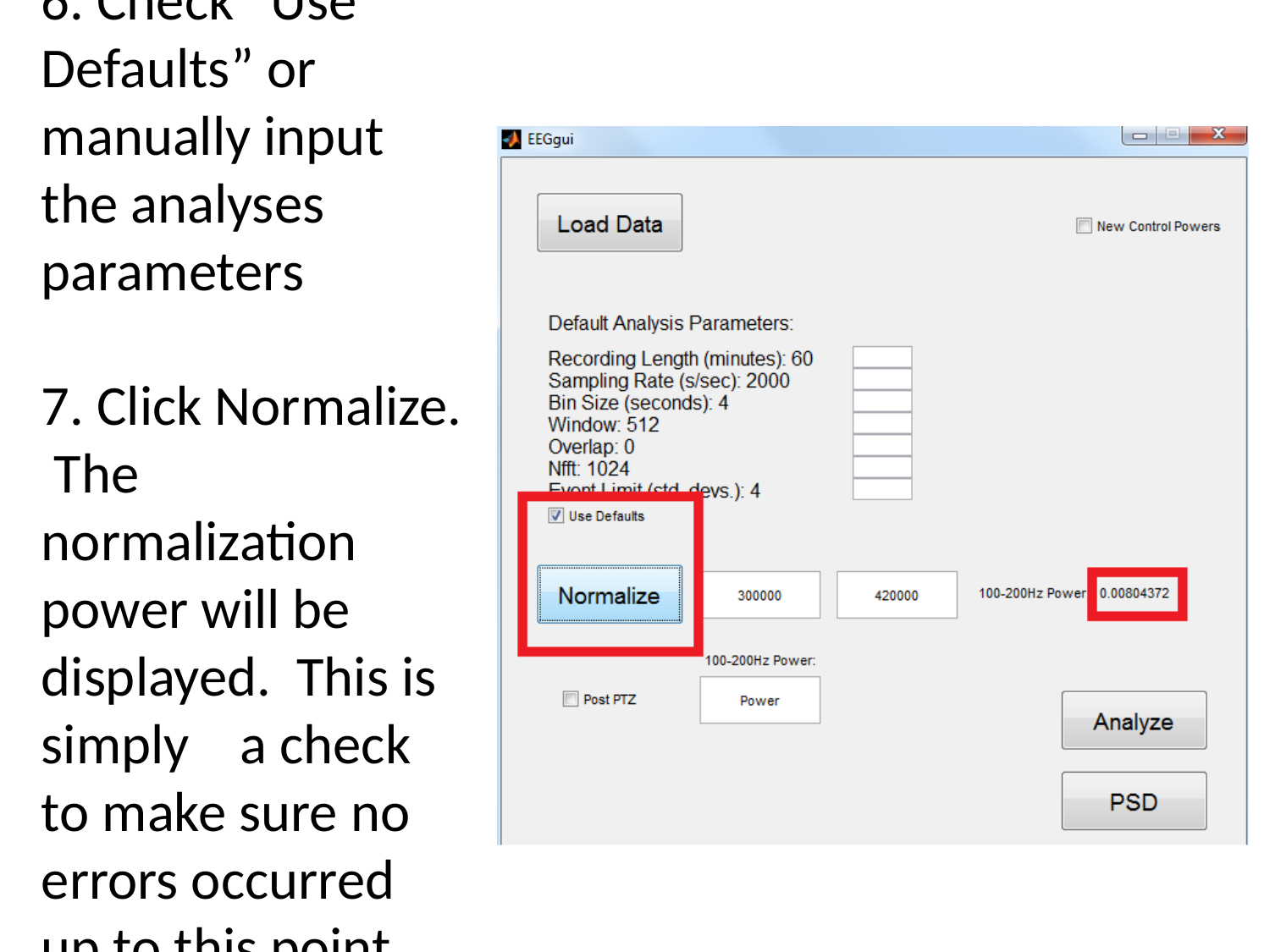

# 6. Check “Use Defaults” or manually input the analyses parameters 7. Click Normalize. The normalization power will be displayed. This is simply a check to make sure no errors occurred up to this point

## Slide 10
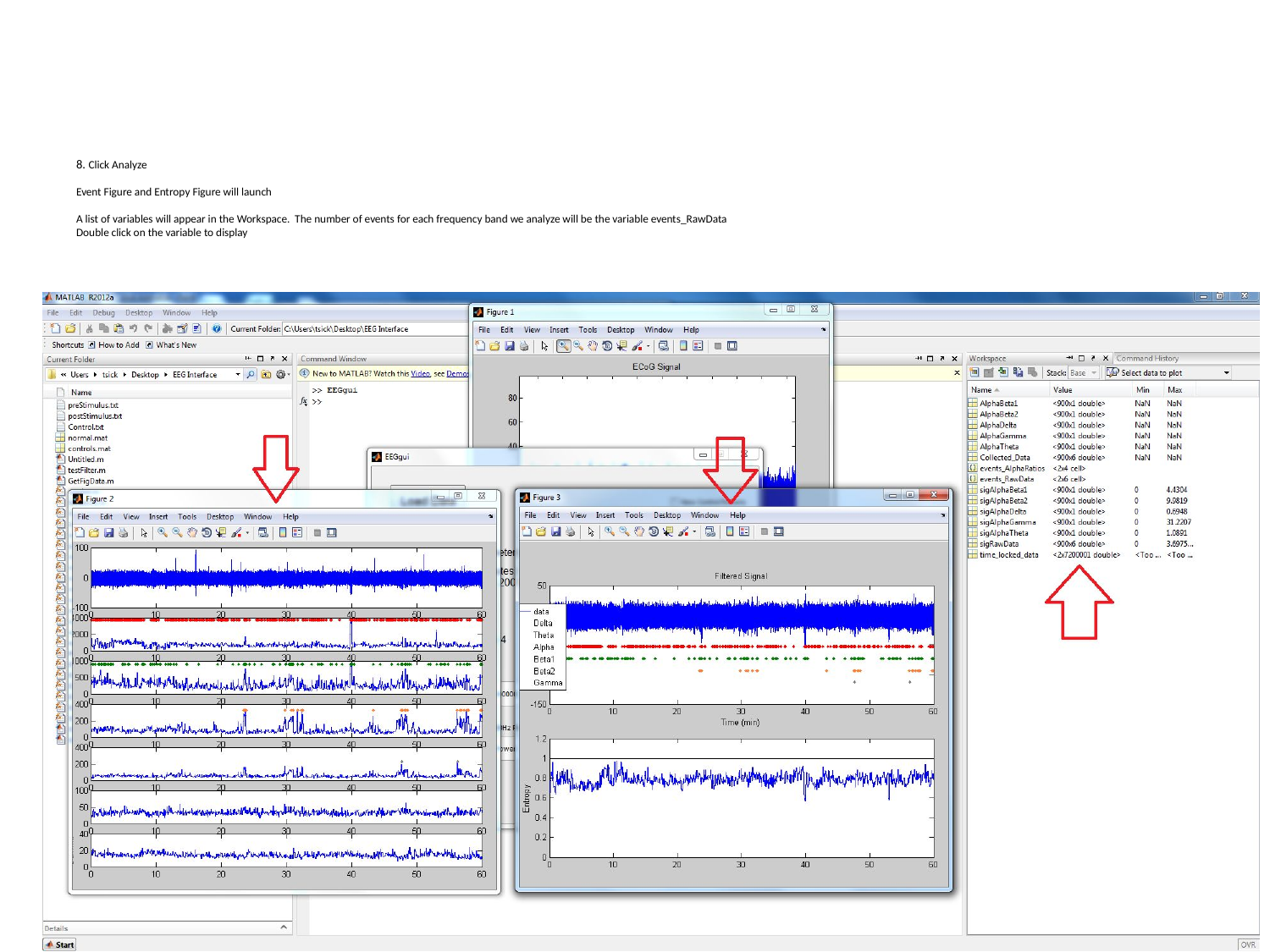

# 8. Click Analyze Event Figure and Entropy Figure will launch A list of variables will appear in the Workspace. The number of events for each frequency band we analyze will be the variable events_RawData Double click on the variable to display

## Slide 11
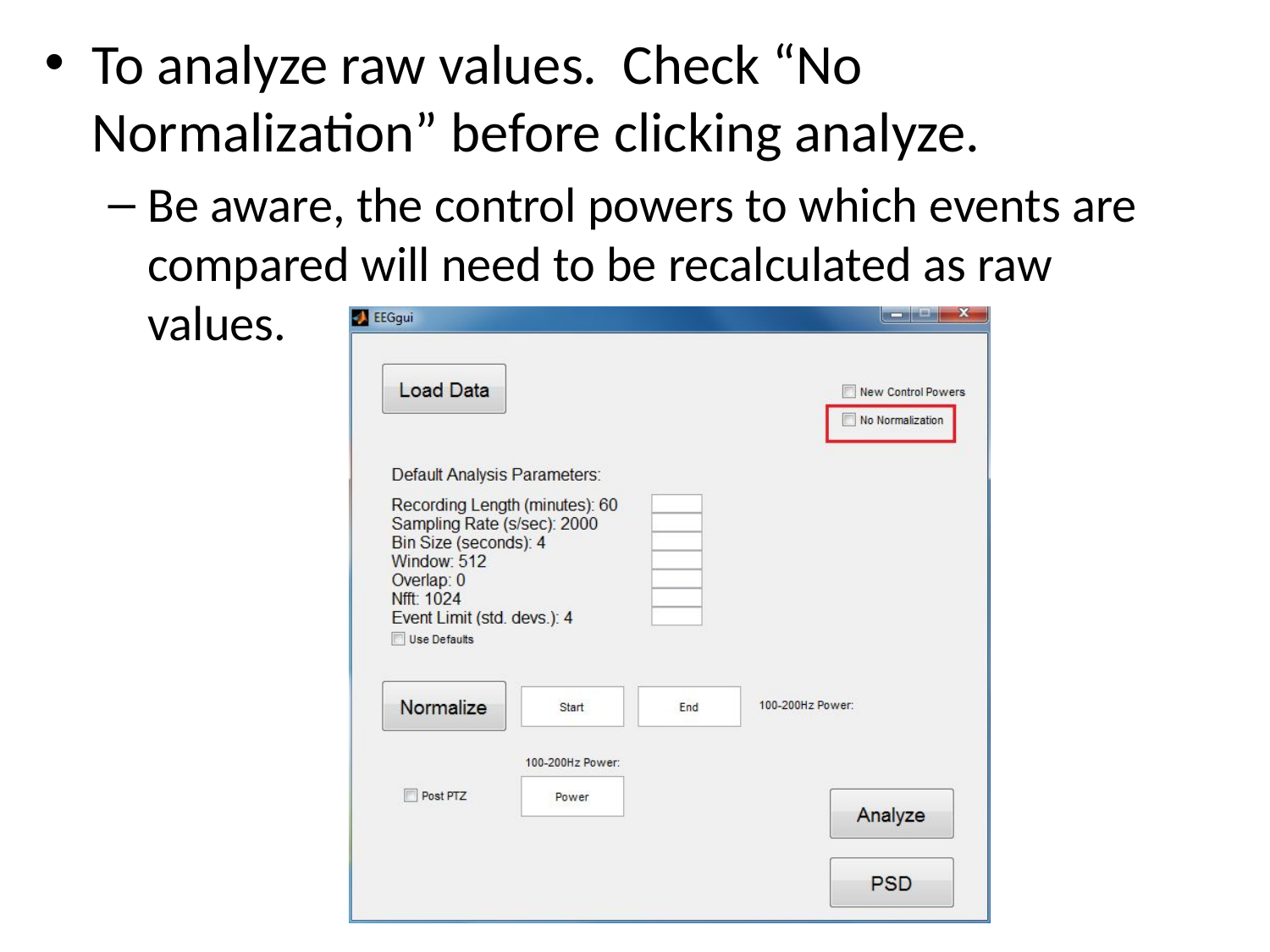

To analyze raw values. Check “No Normalization” before clicking analyze.
Be aware, the control powers to which events are compared will need to be recalculated as raw values.
